# Morbidity and risk factors of COVID‐19 in people with HIV‐1 in Europe: A combined observational cohort and nested case–control study

**DOI:** 10.1111/hiv.70094

**Published:** 2025-08-07

**Authors:** Georg M. N. Behrens, Lambert Assoumou, Stephane De Wit, Rona MacDonald, Nathalie de Castro, Casper Rokx, Holly Middleditch, Margaret Johnson, Jose Luis Casado, Jose Ramon Arribas, Jose‐Ramon Blanco, Carl Fletcher, Caroline Eteve‐Pitsaer, Aliou Baldé, Anton Pozniak, Esteban Martinez, the HIV CoCo Study Group, Arkaitz Imaz, David Rial Crestelo, Mar Masia Canuto, Jose Luis Blanco Arevalo, Alberto Díaz De Santiago, Pere Domingo Pedrol, Patrick Philibert, Jean‐Michel Molina, Frank Post, Ade Apoola, Chloe Orkin, Mark Gompels, Margherita Bracchi, Julie Fox, Marta Vasylyev

**Affiliations:** ^1^ Department of Rheumatology and Immunology Hannover Medical School Hanover Germany; ^2^ Sorbonne Université, INSERM, Institut Pierre Louis d'Epidémiologie et de Santé Publique Paris France; ^3^ CHU St Pierre Brussels Belgium; ^4^ Brownlee Centre, Gartnavel General Hospital Glasgow UK; ^5^ Hopital Saint Louis Paris France; ^6^ Department of Internal Medicine and Department of Medical Microbiology and Infectious Diseases Erasmus Medical Centre Rotterdam The Netherlands; ^7^ King's College Hospital London UK; ^8^ Royal Free Hospital London UK; ^9^ Hospital Universitario Ramon y Cajal Madrid Spain; ^10^ Hospital Universitario La Paz Madrid Spain; ^11^ Hospital Universitario San Pedro Logroño Spain; ^12^ Research Organisation Kings Cross London UK; ^13^ Cegedim Health Data Boulogne‐Billancourt France; ^14^ Chelsea and Westminster Hospital London UK; ^15^ LSHTM London UK; ^16^ The European Treatment Network for HIV, Hepatitis and Global Infectious Diseases (NEAT ID), Belgium Brussels Belgium; ^17^ Hospital Clínic Barcelona Spain; ^18^ CIBER de Enfermedades Infecciosas (CIBERINFEC), Instituto de Salud Carlos III Madrid Spain

**Keywords:** comorbidity, COVID‐19, Europe, HIV infection, risk

## Abstract

**Objective:**

To study the COVID‐19 disease course in people living with HIV (PLWH) based on meaningful case numbers, information about comorbidities, antiretroviral and COVID‐19 treatment and HIV disease‐related variables.

**Methods:**

Multi‐centre, observational, retrospective study of people living with HIV with COVID‐19 matched to HIV‐uninfected individuals with COVID‐19 (HUC) and a case–control study of people living with HIV with COVID‐19 matched to COVID‐19 negative people living with HIV. Kaplan–Meier estimates and Cox proportional‐hazards models stratified on each matched pair were used for COVID‐19 outcomes, and conditional logistic regression models were used to identify risk factors for COVID‐19 infection.

**Results:**

Five hundred people living with HIV and COVID‐19, 1106 HUC and 992 people living with HIV without COVID‐19 were included. Rates for chronic kidney disease, peripheral vascular disease, dementia, prior pneumonia and liver disease in people living with HIV with COVID‐19 were significantly higher by 4.1‐, 2.9‐, 2.6‐, 2.4‐ and 1.6‐fold, respectively, compared to HIV seronegative COVID‐19 patients. Chronic kidney disease, chronic obstructive pulmonary disease (COPD), body mass index (BMI) ≥30 kg/m^2^, Centers for Disease Control and Prevention stage B versus A and HIV viral load ≥50 copies/mL were significant risk factors for COVID‐19 in people living with HIV. Critical care admission or death in people living with HIV was comparable to HUC, whilst people living with HIV stayed longer in hospital (11 vs. 9 days) and in intensive care unit (ICU) (18 vs. 7 days) and had a higher age‐adjusted Charlson comorbidity index.

**Conclusions:**

Our study highlights the importance of a well‐controlled HIV infection to prevent severe COVID‐19‐related outcomes. In people living with HIV and COVID‐19, chronic kidney disease and a higher Charlson comorbidity index are risk factors that should prompt early treatment of COVID‐19.

## INTRODUCTION

The COVID‐19 pandemic was a public health emergency of international concern with mortality rates of 2%–4% and the highest death risk in elderly male patients and those with comorbidities including arterial hypertension, cardiovascular disease, chronic lung disease, obesity and diabetes. Preliminary data reported the disease course of COVID‐19 in specific patient groups with immunosuppression or people living with HIV [1, 2, 3, 4] and some suggested that people living with HIV may have some degree of protection due to their antiretroviral therapy (ART) compounds [5, 6, 7, 8]. Irrespective of their ART, people living with HIV with comorbidities were expected to have risk factors for poorer COVID‐19 outcomes, since cardiovascular and chronic lung disease are more common in this population and almost half of people living with HIV in Europe are older than 50 years. Also, incomplete immune reconstitution may put them at risk for severe COVID‐19.

Case series of people living with HIV with COVID‐19 from China, Spain, Germany, Italy and the United States [3, 9, 10, 11, 12, 13, 14, 15, 16] showed no clear evidence for a higher SARS‐CoV‐2 infection rate or different disease course in people with and without HIV. Of note, most case series of people living with HIV reported a younger age in their study population than in HIV‐negative hospitalized COVID‐19 patients, but comparable rates of comorbidities.

Among the larger studies, a retrospective cohort of electronic medical record data in the United States found that people living with HIV and more advanced HIV disease (greater immunosuppression and HIV viraemia) were at higher risk for severe COVID‐19 outcomes [17]. An analysis, in which outcomes in COVID‐19 positive people living with HIV were compared with a propensity‐matched cohort of patients without HIV, found that the crude COVID‐19 mortality was higher in people living with HIV. However, propensity‐matched analyses revealed no difference in outcomes, showing that higher mortality is driven by a higher burden of comorbidities [18]. A cohort study from the United Kingdom reported a potential increase in the risk of mortality among people living with HIV under 60 years of age once hospitalized with COVID‐19. Mortality was higher among people living with HIV after adjusting for age (adjusted hazard ratio 1.47, 95% confidence interval [CI], 1.01–2.14; *p* = 0.05), and the association persisted after adjusting for the other variables and when restricting the analysis to people aged <60 years. However, the study provided no data around the risk of developing severe COVID‐19 or hospitalization among this cohort of 112 people living with HIV and no data on viral load or CD4 count [19]. A retrospective study from Spain with 749 people living with HIV and COVID‐19 found that SARS‐CoV‐2 diagnosis was more common among migrants, men who have sex with men and those with four or more chronic comorbidities. Age above 75 years, non‐Spanish origin and neuropsychiatric, autoimmune disease, respiratory disease and metabolic disease chronic comorbidities were associated with an increased risk of severe outcomes [20]. An analysis of risk factors for COVID‐19 deaths in the Western Cape described that after adjusting for other risk factors, HIV increased a COVID‐19 patient's death risk by a factor of 2.14 (95% CI, 1.70–2.70) [21].

A meta‐analysis [22] concluded that people living with HIV and HIV‐negative individuals showed comparable rates and intensity of COVID‐19. ART users exhibited immunological health comparable to immunocompetent people, demonstrating the essential role of antiretroviral therapy in reducing the severity and mortality of people living with HIV with COVID‐19. Another narrative review [23] reported that out of 57 studies assessing risk factors and clinical outcomes in HIV patients co‐infected with COVID‐19, 39 of the studies indicated several comorbidities being associated with severe outcomes in HIV and COVID‐19, whilst 20 studies reported no difference in clinical outcomes.

To date, many studies assessing the risk factors for COVID‐19 and clinical outcomes in people living with HIV were restricted by the number of cases (mostly 100–200 people living with HIV and COVID‐19), were solely created from medical record databases or included only hospitalized patients, were deficient in completeness of data on comorbidities and biomedical data, frequently lacked information on antiretroviral or COVID‐19 treatment, failed to include HIV disease‐related parameters and had clear limitations when comparing people living with HIV without COVID‐19 as control groups. To overcome these limitations, we performed an observational, retrospective study with 500 people living with HIV and COVID‐19 recruited from multiple European sites and two control groups with 2098 patients to simultaneously evaluate the severity of COVID‐19 in people living with HIV as compared to control COVID‐19 cases without HIV as well as to describe risk factors for COVID‐19 within the group of people living with HIV.

## MATERIALS AND METHODS

The HIV COVID‐19 Co‐Infection (HIV CoCo) Study is an observational, retrospective combined exposure/non‐exposure and case–control study. The study population consisted of adult people living with HIV with and without COVID‐19 disease followed up at 21 study sites in five countries (UK, Spain, France, Belgium and the Netherlands) for routine clinical care. In addition, data from adult HIV‐uninfected COVID‐19 patients were used as controls. People living with HIV and COVID‐19 were identified from sites in the NEAT ID network after launching a dashboard in March 2020, in which 50 European sites registered to provide basic information about the number of people living with HIV with COVID‐19 and disease outcomes (https://www.NEAT-ID.org/). Ambulatory HUC were identified from COVID‐19 outpatients from THIN® France (The Health Improvement Network), a European clinical database network of Electronic Health Records extracts. These extracts are transmitted in compliance with current regulations, including General Data Protection Regulation (GDPR) by a network of voluntary physicians (general practitioners and specialists, https://www.the‐health‐improvement‐network.com). Hospitalized HUC were identified from NEAT ID network sites.

Cases (people living with HIV and COVID‐19) were at least 18 years of age with documented HIV‐1 infection and confirmed COVID‐19 by documented, or patient‐reported, positive result on polymerase chein reaction (PCR) testing of a nasopharyngeal or respiratory sample, before 1 April 2021 when COVID‐19 vaccination became available. HUC were at least 18 years of age without documented HIV‐1 infection but confirmed COVID‐19 by documented, or patient‐reported, positive result on PCR testing of a nasopharyngeal or respiratory sample, before 1 April 2021. The second control group (people living with HIV without COVID‐19) included individuals at least 18 years of age with documented HIV‐1 infection and no COVID‐19.

For comparing people living with HIV with COVID‐19 versus HUC, we performed a 1:1 and 1:3 matching according to the following criteria: age (±5 years), sex, ethnicity (where available), month of COVID‐19 diagnosis (±2 months) and diagnosis as inpatient or outpatient. Inpatients with COVID‐19 diagnosis were matched 1:1; ambulatory patients with COVID‐19 were matched at 1:3. For comparing people living with HIV and COVID‐19 versus people living with HIV without COVID‐19, individuals were matched at 1:2 for similar risk of acquiring COVID‐19 according to the following criteria: age (±5 years), sex, ethnicity (where available).

### Data collection

For all patients, we collected age, sex, ethnicity (where available), comorbidities (including myocardial infarction, congestive heart failure, peripheral vascular disease, cerebrovascular accident or transient ischaemic attack, dementia, chronic obstructive pulmonary disease [COPD], history of pneumonia, connective tissue disease, peptic ulcer disease, liver disease, diabetes, hemiplegia, paralysis of arm[s] or leg[s], chronic kidney disease [CKD], current or history of cancer, leukaemia, lymphoma, AIDS [where applicable]), body mass index (BMI), Charlson Comorbidity Index (CCI) and age‐adjusted CCI (ACCI). For people living with HIV, the following additional data were collected: date of HIV diagnosis, current antiretroviral therapy, Centers for Disease Control and Prevention (CDC) disease stage, CD4 T lymphocyte nadir, last (≤6 months) CD4 T cell count (including CD4 T cell percentage and CD4/CD8 ratio) and last HIV‐RNA before COVID‐19 diagnosis, or most recent (≤6 months). For patients with COVID‐19, the following additional information was collected, if available: date of positive PCR test result (documented or patient‐reported), COVID‐19 diagnosis inpatient or outpatient, all‐cause mortality in hospital or palliative discharge, or mortality at 6 weeks after diagnosis of COVID‐19 or at discharge from hospital, hospitalization for COVID‐19 and length of hospitalization, critical care admission (high dependency unit or intensive care unit) for COVID‐19 and length of stay in critical care, length of invasive ventilation (number of ventilator‐free days), length of extracorporeal membrane oxygenation (ECMO), need for kidney replacement therapy, laboratory markers and blood cell counts at COVID‐19 diagnosis (e.g., Alanine‐aminotransferase, Aspartate‐aminotransferase (AST), serum creatinine, calcium, glucose and HbA1c levels, C‐reactive protein [CRP], D‐dimer and lactate dehydrogenase) and drug treatment for COVID‐19. Real‐world retrospective data from people living with HIV and from hospitalized HUC were transcribed into the eCRF system from source data at each clinical site. Data from ambulatory HUC were obtained from THIN® France, a network of longitudinal databases covering over 35 million electronic health records.

### Outcomes

The primary outcomes of our study were (1) the proportion of participants with a severe form of COVID‐19 infection (critical care admission [high dependency unit or intensive care unit], or palliative discharge when discharged from hospital, or mortality within the 6 weeks after diagnosis of COVID‐19) in people living with HIV in comparison to HUC, and (2) identifying risk factors (e.g., CD4 T cell nadir, current CD4 T cell count, comorbidities) for COVID‐19 infection within the group of people living with HIV.

### Statistical analysis

For the sample size calculation and statistical analysis, please see Supplementary material.

## RESULTS

### Objective 1: Outcomes of COVID‐19 in people living with HIV in comparison to HIV‐uninfected COVID‐19 controls

In total, we included 500 people living with HIV and COVID‐19, 1106 HUC with COVID‐19 and 992 people living with HIV without COVID‐19. Fourteen inpatient people living with HIV with COVID‐19 had no control. The key baseline demographics and clinical characteristics are listed in Table 1, which also contains information about other matching criteria.

**TABLE 1:** Baseline demographics and clinical characteristics.

Characteristics	Participants		*p*‐Value^b^
	PLWH with COVID‐19, *N* = 486^a^	HUC with COVID‐19, *N* = 1106	
Age (years)			
*N*	486	1106	
Median (IQR)	51 (43–58)	51 (43–58)	
[Range]	[21–89]	[21–88]	
Mean (SD)	51 (11.2)	51 (11.1)	
*N* (%)			
<60 years	378 (77.8)	878 (79.4)	
≥60 years	108 (22.2)	228 (20.6)	
Gender, *n* (%)			
Female	171 (35.2)	409 (37.0)	
Male	315 (64.8)	697 (63.0)	
Ethnicity, *n* (%)			**0.041**
*N*	344	344	
White Caucasian	150 (43.6)	148 (43.0)	
White mixed	19 (5.5)	24 (7.0)	
Asian	4 (1.2)	4 (1.2)	
Black	105 (30.5)	86 (25.0)	
Other	8 (2.3)	4 (1.2)	
Not stated	58 (16.9)	78 (22.7)	
Month of COVID‐19 diagnosis			
Median (IQR)	10/2020 (04/2020–12/2020)	10/2020 (04/2020–12/2020)	
[Range]	[01/2020–03/2021]	[01/2020–03/2021]	
COVID‐19 diagnosis location, *n* (%)			
Inpatient	176 (36.2)	176 (15.9)	
Outpatient (ambulatory)	310 (63.8)	930 (84.1)	
Body weight (kg)			0.272
*N*	365	779	
Median (IQR)	80 (70–91)	80 (68–92)	
[Range]	[38–200]	[10–184]	
Mean (SD)	82 (18.0)	81 (19.1)	
BMI (kg/m^2^)			**0.048**
*N*	305	581	
Median (IQR)	27.0 (24.2–31.2)	28.0 (24.6–31.5)	
[Range]	[14.8–69.2]	[14.7–61.3]	
Mean (SD)	28.1 (6.4)	28.7 (6.2)	
*N* (%)			0.112
<30 kg/m^2^	212 (69.5)	376 (64.7)	
≥30 kg/m^2^	93 (30.5)	205 (35.3)	

*Note*: Bold indicates statistically significant values (*p* < 0.05).

Abbreviations: BMI, body mass index; HUC, HIV‐uninfected controls; IQR, interquartile range; PLWH, people living with HIV.

^a^ *N* = 14/500 participants had no matched controls.

^b^ *p*‐Value calculated using McNemar test for categorical variable and Wilcoxon paired test for continuous variables.

The 486 people living with HIV with COVID‐19 had a median age of 51 years (interquartile range, IQR 15); 108 (22.2%) were ≥60 years, 315 (64.8%) were male, 150/344 (43.6%) were white Caucasians and their median BMI was 27.0 kg/m^2^ (IQR 24.2–31.2).

People living with HIV and COVID‐19 compared to HUC had higher rates of CKD (9.1 vs. 2.2%, *p* < 0.001), peripheral vascular disease (7.5% vs. 2.6%, *p* = 0.006), dementia (4.4% vs. 1.7%, *p* = 0.039), history of pneumonia (17.7% vs. 7.3%, *p* < 0.001) and liver disease (13.7% vs. 8.5%, *p* = 0.003), and lower rates of connective tissue disease (4.3% vs. 22.7%, *p* < 0.001) and diabetes (12.7% vs. 20.7%, *p* < 0.001, Table 2).

**TABLE 2:** Baseline comorbidities of PLWH with COVID‐19 versus PLWH without COVID‐19 (left) and of PLWH with COVID‐19 versus COVID‐19 patients without HIV (right).

Comorbidities, *n*/*N* (%)	PLWH with COVID‐19, *n* = 500	PLWH without COVID‐19, *n* = 992	*p*‐Value^a^	PLWH with COVID‐19, *n* = 486	HUC with COVID‐19, *n* = 1106	*p*‐Value^b^
Myocardial infarction	16/500 (3.2)	34/992 (3.4)	0.835	15/395 (3.8)	17/634 (2.7)	0.537
Congestive heart failure	13/500 (2.6)	20/992 (2.0)	0.455	12/395 (3.0)	21/634 (3.3)	0.544
Peripheral vascular disease	28/500 (5.6)	42/992 (4.2)	0.212	24/320 (7.5)	9/344 (2.6)	**0.006**
Stroke or transient ischaemic attack	19/500 (3.8)	25/992 (2.5)	0.203	14/395 (3.5)	24/634 (3.8)	0.670
Dementia	16/500 (3.2)	19/992 (1.9)	0.129	14/320 (4.4)	6/344 (1.7)	**0.039**
COPD	36/500 (7.2)	43/992 (4.3)	**0.012**	31/395 (7.9)	35/634 (5.5)	0.143
History of pneumonia	83/500 (16.6)	146/992 (14.7)	0.339	70/395 (17.7)	46/634 (7.3)	**<0.001**
Connective tissue disease	21/500 (4.2)	25/992 (2.5)	0.957	17/395 (4.3)	144/634 (22.7)	**<0.001**
Peptic ulcer disease	21/500 (4.2)	35/992 (3.5)	0.461	16/395 (4.1)	30/634 (4.7)	0.959
Liver disease	69/500 (13.8)	141/992 (14.2)	0.843	54/395 (13.7)	54/634 (8.5)	**0.003**
Diabetes	59/500 (11.8)	97/992 (9.8)	0.226	50/395 (12.7)	131/634 (20.7)	**<0.001**
Hemiplegia	2/500 (0.4)	3/992 (0.3)	0.753	2/320 (0.6)	0/344 (0.0)	0.987
Paralysis of arm(s) or leg(s)	3/500 (0.6)	3/992 (0.3)	0.396	3/395 (0.8)	6/634 (1.0)	0.533
Chronic kidney disease	38/500 (7.6)	32/992 (3.2)	**<0.001**	36/395 (9.1)	14/634 (2.2)	**<0.001**
Current or history of cancer	46/500 (9.2)	99/992 (10.0)	0.620	42/395 (10.6)	79/634 (12.5)	0.162
Leukaemia	1/500 (0.2)	3/992 (0.3)	0.726	1/395 (0.3)	3/634 (0.5)	0.504
Lymphoma	12/500 (2.4)	30/992 (3.0)	0.514	10/395 (2.5)	10/634 (1.6)	0.485

*Note*: Bold indicates statistically significant values (*p* < 0.05).

Abbreviations: COPD, chronic obstructive pulmonary disease; HUC, HIV‐uninfected controls; PLWH, people living with HIV.

^a^ *p*‐Value calculated using Conditional logistic regression model.

^b^ *p*‐Value calculated using McNemar test for categorical variable.

People living with HIV and COVID‐19 had significantly lower median values for red blood cell counts (*p* = 0.002), lactate dehydrogenase (*p* = 0.038) and AST (*p* = 0.009) but their median values for mean corpuscular volume (MCV, *p* < 0.001), mean corpuscular haemoglobin (MCH, *p* < 0.001), lymphocyte counts (*p* < 0.001), serum creatinine (*p* = 0.020) and total cholesterol (*p* = 0.041) were higher (Table S1). People living with HIV and COVID‐19 were more often treated with hydroxychloroquine (49.2% vs. 38.0%, *p* = 0.007), but less often with glucocorticoids (29.4% vs. 48.6%, *p* = 0.002), anti‐IL 6 inhibitors (6.4% vs. 15.5%, *p* = 0.010) or other experimental treatments (16.5% vs. 30.3%, *p* = 0.008, Table S2).

**FIGURE 1:**
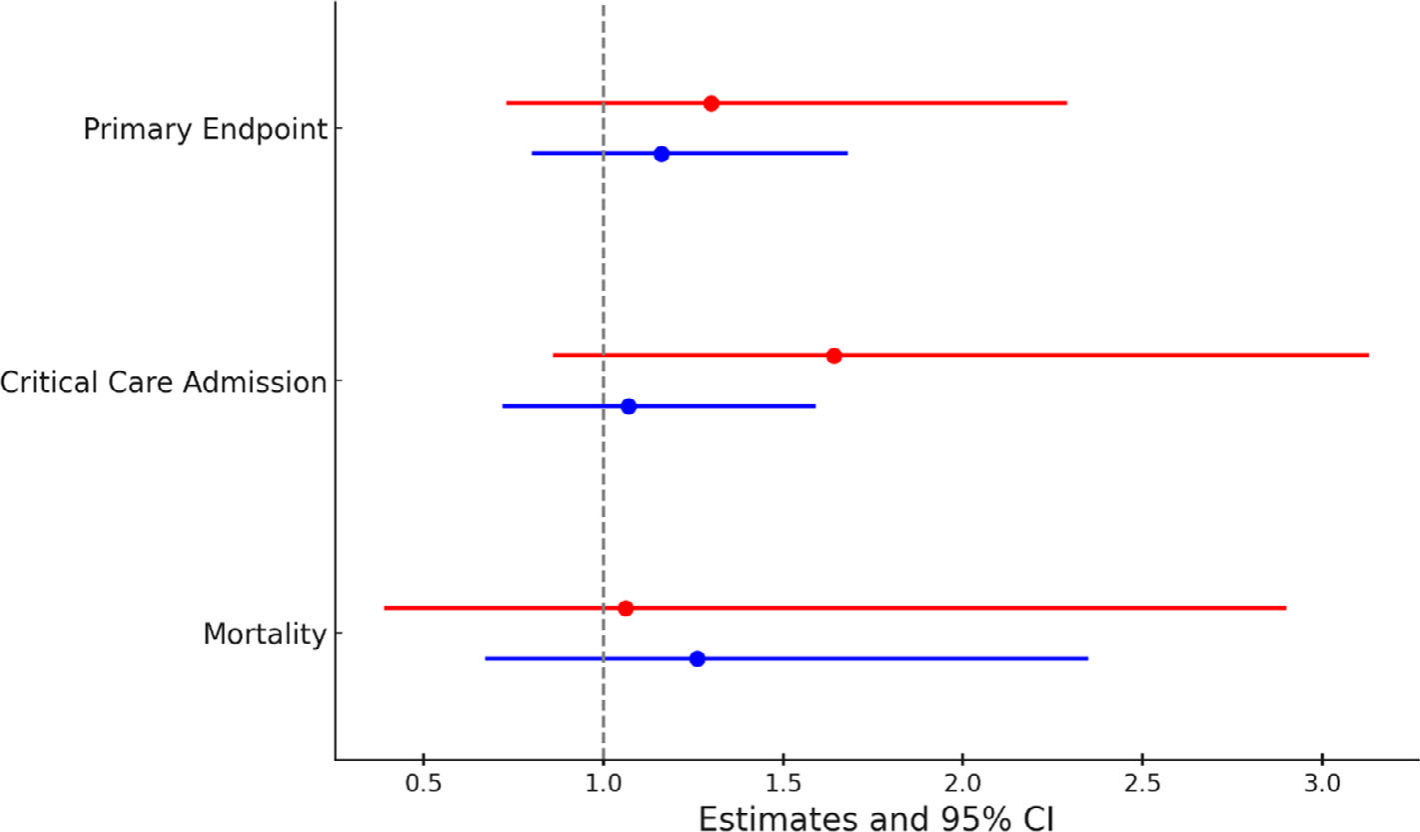
Primary and secondary outcomes of people living with HIV with COVID‐19 versus COVID‐19. Depicted are the crude (blue) and adjusted measures (red) of association (95% confidence interval [CI]) for the composite primary endpoint (critical care admission, palliative discharge, death), critical care admission and mortality. For adjusted factors, see Table 3.

Results for the composite primary endpoint and secondary endpoints are listed in Table 3 and depicted in Figure 1. The absolute Kaplan–Meier estimate of the primary endpoint was 13.6% in people living with HIV and COVID‐19 and 6.1% in HUC and COVID‐19, with an adjusted HR (aHR) of 1.30 (95% CI, 0.73–2.29). The same applies to the risk of mortality 6 weeks after COVID‐19 (aHR 1.06; 95% CI, 0.39–2.90) and admission to critical care (aHR 1.64; 95% CI, 0.86–3.13). Only one people living with HIV with COVID‐19 received palliative discharge, and none in the HUC and COVID‐19 group. The adjusted sub‐distribution HR (sHR) for cumulative incidence for discharge from the hospital was 0.63; 95% CI, 0.48–0.84, and cumulative incidence for discharge from ICU was 0.06; 95% CI, 0.01–0.34. This means that the median length of hospital and ICU stay was longer in people living with HIV and COVID‐19 than in HUC. Interestingly, the age‐adjusted CCI was significantly higher in people living with HIV and COVID‐19 than in HUC (adjusted mean difference 1.62; 95% CI, 1.14–2.22), as were differences in mean MCV (4.30; 95% CI, 2.77–5.85) and mean MCH (1.55; 95% CI, 1.09–2.01). The need for kidney replacement therapy had an adjusted odds ratio (aOR) of 1.81; 95% CI, 0.54–6.02 (Table S3).

**TABLE 3:** Primary and secondary outcomes of PLWH with COVID‐19 versus COVID‐19.

	PLWH with COVID‐19, *N* = 486	HUC and COVID‐19, *N* = 1106	Crude measure of association (95% CI)	Adjusted measure of association (95% CI)^a^
Number of composite primary endpoint (critical care admission, palliative discharge, death) events	66	67		
Kaplan–Meier estimate of primary endpoint—% (95% CI)	13.6 (10.8–17.0)	6.1 (4.8–7.6)	1.16 (0.80–1.68)	1.30 (0.73–2.29)
Number of critical care admission	55	58		
Kaplan–Meier estimate of critical care admission—% (95% CI)	11.4 (8.9–14.6)	5.3 (4.1–6.8)	1.07 (0.72–1.59)	1.64 (0.86–3.13)
Number of palliative discharge	1	0		
Kaplan–Meier estimate of palliative discharge—% (95% CI)	0.2 (0.1–1.5)	0.0 (0.0–0.3)	n.a.	n.a.
Mortality: number of deaths	24	23		
Kaplan–Meier estimate of deaths—% (95% CI)	4.9 (3.3–7.3)	2.1 (1.4–3.1)	1.26 (0.67–2.35)	1.06 (0.39–2.90)

Abbreviations: CI, confidence interval; HUC, HIV‐uninfected controls; n.a., not applicable; PLWH, people living with HIV.

^a^ Adjusted on body mass index, dementia, peripheral vascular disease, history of pneumonia, connective tissue disease, liver disease, diabetes, chronic kidney disease, glucocorticoids, antibiotics, hydroxychloroquine, tocilizumab, anti‐IL6 inhibitors and other drugs for the treatment of COVID‐19.

In summary, rates of CKD, peripheral vascular disease, dementia, history of pneumonia and liver disease were between 0.6‐ and 3.1‐fold higher in people living with HIV and COVID‐19, whilst rates for diabetes and connective tissue disease were 0.6‐fold and 4.2‐fold lower compared to HUC. In line with this, people living with HIV and COVID‐19 had a higher median AACI, and their relative risk for severe COVID‐19 disease outcome increased by 30%.

### Objective 2: Risk factors for severe COVID‐19 outcomes within the group of people living with HIV

The baseline demographics and clinical characteristics of people living with HIV with COVID‐19 versus people living with HIV without COVID‐19 are listed in Table 4. Compared to people living with HIV and no COVID‐19, the median age, sex and ethnicity (where available) were matching criteria and thus similar. Compared to people living with HIV and no COVID‐19, the median BMI in people living with HIV with COVID‐19 was significantly higher (27.1 kg/m^2^ [IQR 7] vs. 26.1 kg/m^2^ [IQR 6.5], *p* < 0.001), and people living with HIV with COVID‐19 had more comorbidities (Table 2). COPD (7.2% vs. 4.3%, *p* = 0.012) and CKD (7.6% vs. 3.2%, *p* < 0.001) were 1.67‐fold and 2.37‐fold different. People living with HIV and COVID‐19 had a shorter median time since HIV diagnosis (17.1 years [IQR 13.3] vs. 18.3 years [IQR 13.6], *p* = 0.005).

**TABLE 4:** Baseline demographics and clinical characteristics of PLWH with COVID‐19 versus PLWH without COVID‐19.

Characteristic	Participants		*p*‐Value
	PLWH and COVID‐19, *n* = 500	PLWH, no COVID‐19, *n* = 992	
Age (years)			0.971
*N*	500	992	
Median (IQR)	52 (44–59)	53 (44–59)	
[Range]	[21–89]	[21–89]	
Mean (SD)	52 (11.3)	52 (11.2)	
*N* (%)			0.452
<60 years	388 (77.6)	778 (78.4)	
≥60 years	112 (22.4)	214 (21.6)	
Gender, *n* (%)			
Female	176 (35.2)	348 (35.1)	
Male	324 (64.8)	644 (64.9)	
Ethnicity, *n* (%)			0.260
White Caucasian	226 (45.2)	464 (46.8)	
White mixed	37 (7.4)	49 (4.9)	
Asian	4 (0.8)	7 (0.7)	
Black	163 (32.6)	329 (33.2)	
Other	12 (2.4)	23 (2.3)	
Not stated	58 (11.6)	120 (12.1)	
Body weight (kg)			**<0.001**
*N*	479	943	
Median (IQR)	80 (70–90)	78 (68–88)	
[Range]	[38–200]	[40–179]	
Mean (SD)	82 (17.7)	79 (16.1)	
BMI (kg/m^2^)			**<0.001**
*N*	428	847	
Median (IQR)	27.1 (24.3–31.3)	26.1 (23.2–29.7)	
Mean (SD)	28.1 (6.0)	26.8 (5.4)	
*N* (%)			**<0.001**
<30 kg/m^2^	286 (66.8)	649 (76.6)	
≥30 kg/m^2^	142 (33.2)	198 (23.4)	
Time since HIV diagnosis (years)			**0.005**
*N*	492	977	
Median (IQR)	17.1 (10.7–24.5)	18.3 (11.8–25.4)	
Mean (SD)	17.8 (9.5)	18.7 (9.3)	
CD4 cell nadir (cells/mm^3^)			0.303
*N*	468	926	
Median (IQR)	224 (80–364)	230 (110–364)	
Mean (SD)	257 (213.1)	263 (202.8)	
CD4 cell count (cells/mm^3^)			0.204
*N*	489	971	
Median (IQR)	631 (417–827)	632 (446–845)	
Mean (SD)	654 (332.9)	669 (321.9)	
CD4%			0.118
*N*	483	956	
Median (IQR)	32.9 (24.9–40.0)	33.3 (25.6–40.0)	
Mean (SD)	32.0 (12.0)	32.8 (11.3)	
CD4/CD8 ratio			0.817
*N*	430	850	
Median (IQR)	0.86 (0.6–1.29)	0.90 (0.57–1.24)	
Mean (SD)	1.36 (5.8)	1.20 (4.0)	
HIV viral load (copies/mL)			**<0.001**
*N*	492	976	
Median (IQR)	20 (20–40)	20 (20–36)	
Mean (SD)	15 773 (235,013.9)	5531 (83,204)	
			**0.008**
<50	423 (86.0)	878 (90.0)	
≥50	69 (14.0)	98 (10.0)	
CDC disease stage, *n* (%)			0.063
*N*	481	953	
A	100 (20.8)	244 (25.6)	
B	225 (46.8)	413 (43.3)	
C	156 (32.4)	296 (31.1)	
AIDS, *n* (%)			0.378
*N*	500	992	
Yes	96 (19.2)	174 (17.5)	
Current ART, *N* (%)			0.999
*N*	493	977	
BIC + FTC + TAF	73 (14.8)	146 (14.9)	
DTG + 3TC + ABC	67 (13.6)	96 (9.8)	
DTG + 3TC	62 (12.6)	131 (13.4)	
DRVc + FTC + TAF	31 (6.3)	67 (6.9)	
RPV + FTC + TAF	26 (5.3)	32 (3.3)	
FTC + TDF	21 (4.3)	55 (5.6)	
DTG	20 (4.1)	28 (2.9)	
3TC + ABC	18 (3.6)	39 (4.0)	
RPV + FTC + TDF	17 (3.5)	34 (3.5)	
EFV + FTC + TDF	14 (2.8)	45 (4.6)	
EVGc + FTC + TAF	13 (2.6)	31 (3.2)	
DTG + RPV	12 (2.4)	31 (3.2)	
Other	119 (24.1)	242 (24.8)	
			0.0596
TDF/TAF‐based regimen	230 (46.7)	505 (51.7)	
Other regimen	263 (53.3)	472 (48.3)	

*Note*: Bold indicates statistically significant values (*p* < 0.05). *p*‐Value calculated using Conditional logistic regression model.

Abbreviations: AIDS, acquired immune deficiency syndrome; ART, antiretroviral therapy; BMI, body mass index; CDC, Centers for Disease Control and Prevention; IQR, interquartile range; PLWH, people living with HIV.

Whilst CD4 T cell counts, CD4/CD8 T cell ratio, HIV CDC disease stage and compositions of antiretroviral therapy regimens were not different between the groups, the proportion of people living with HIV and COVID‐19 with detectable HIV‐RNA was significantly higher (14% vs. 10%, *p* = 0.008).

Using a multivariable analysis, we identified the following significant risk factors for COVID‐19 in people living with HIV: CKD (aOR 2.19; 95% CI, 1.28–3.74, *p* = 0.004), COPD (aOR 1.83; 95% CI, 1.07–3.11, *p* = 0.027), BMI ≥30 kg/m^2^ (aOR 1.73; 95% CI, 1.32–2.26, *p* < 0.001), CDC stage B versus A (aOR 1.51; 95% CI, 1.09–2.10, *p* = 0.042) and HIV‐RNA above 50 copies/mL (aOR 1.52; 95% CI, 1.01–2.29 [*p* = 0.045]) (Table 5).

**TABLE 5:** Factors associated with COVID‐19 infection in PLWH.

Characteristic	Participants		Conditional logistical regression model			
			Univariable analysis		Multivariable analysis	
	PLWH with COVID‐19, *N* = 500	PLWH without COVID‐19, *N* = 992	OR (95% CI)	*p*‐Value	OR (95% CI)	*p*‐Value
Age (years)				0.451		
<60	388 (77.6)	778 (78.4)	1			
≥60	112 (22.4)	214 (21.6)	1.31 (0.65–2.61)			
Gender, *n* (%)						
Female	176 (35.2)	348 (35.1)				
Male	324 (64.8)	644 (64.9)				
Ethnicity, *n* (%)						
White Caucasian	226 (45.2)	464 (46.8)	1	0.260		
White mixed	37 (7.4)	49 (4.9)	2.41 (1.01–5.75)			
Asian	4 (0.8)	7 (0.7)	1.31 (011–15.4)			
Black	163 (32.6)	329 (33.2)	1.07 (0.28–4.11)			
Other	12 (2.4)	23 (2.3)	1.17 (0.15–9.43)			
Not stated	58 (11.6)	120 (12.1)	0.65 (0.11–3.67)			
Body weight (kg), median (IQR)				**<0.001**		
	80 (70–91)	77 (68–88)	1.74 (1.34–2.25)			
BMI (kg/m^2^)				**<0.001**		**<0.001**
<30 kg/m^2^	336 (67.1)	756 (76.2)	1		1	
≥30 kg/m^2^	164 (32.9)	236 (23.8)	1.68 (1.29–2.19)		1.73 (1.32–2.26)	
Comorbidities, *n* (%)						
Myocardial infarction (heart attack)	16 (3.2)	34 (3.4)	0.94 (0.5–1.75)	0.835		
Congestive heart failure	13 (2.6)	20 (2.0)	1.31 (0.65–2.67)	0.455		
Peripheral vascular disease	28 (5.6)	42 (4.2)	1.41 (0.82–2.40)	0.212		
Cerebrovascular accident (stroke) or transient ischaemic attack	19 (3.8)	25 (2.5)	1.51 (0.80–2.85)	0.203		
Dementia	16 (3.2)	19 (1.9)	1.71 (0.86–3.41)	0.129	1.65 (0.80–3.39)	0.178
Chronic obstructive pulmonary disease	36 (7.2)	43 (4.3)	1.93 (1.16–3.22)	**0.012**	1.83 (1.07–3.11)	**0.027**
History of pneumonia	83 (16.6)	146 (14.7)	1.16 (0.85–1.58)	0.339		
Connective tissue disease	21 (4.2)	25 (2.5)	1.69 (0.91–3.13)	0.957		
Peptic ulcer disease	21 (4.2)	35 (3.5)	1.26 (0.68–2.31)	0.461		
Liver disease	69 (13.8)	141 (14.2)	0.97 (0.69–1.35)	0.843		
Diabetes	59 (11.8)	97 (9.8)	1.25 (0.87–1.79)	0.226		
Hemiplegia	2 (0.4)	3 (0.3)	1.33 (0.22–7.98)	0.753		
Paralysis of arm(s) or leg(s)	3 (0.6)	3 (0.3)	2.00 (0.40–9.91)	0.396		
Chronic kidney disease	38 (7.6)	32 (3.2)	2.71 (1.61–4.57)	**<0.001**	2.19 (1.28–3.74)	**0.004**
Current or history of cancer	46 (9.2)	99 (10.0)	0.91 (0.61–1.34)	0.620		
Leukaemia	1 (0.2)	3 (0.3)	0.67 (0.07–6.41)	0.726		
Lymphoma	12 (2.4)	30 (3.0)	0.80 (0.41–1.56)	0.514		
Time since HIV diagnosis						
Per 10 unit for the model	17.1 (10.7–24.6)	18.3 (11.8–25.3)	0.85 (0.74–0.99)	**0.033**	0.86 (0.74–1.00)	0.057
CD4 cell nadir						
Per 100 unit for the model	224 (81–364)	226 (107–360)	0.98 (0.93–1.04)	0.458		
CD4 cell count						
Per 100 unit for the model	630 (414–827)	630 (443–840)	0.99 (0.95–1.02)	0.453		
CD4%						
Per 100 unit for the model	32.9 (25.0–40.0)	33.1 (25.0–40.0)	0.95 (0.86–1.05)	0.296		
CD4/CD8 ratio	0.86 (0.52–1.31)	0.89 (0.54–1.28)	1.00 (0.98–1.02)	0.734		
HIV viral load (copies/mL)				**0.009**		**0.045**
<50	427 (85.4)	888 (89.5)	1		1	
≥50	73 (14.6)	104 (10.5)	1.67 (1.13–2.45)		1.52 (1.01–2.29)	
CDC disease stage, *n* (%)				0.051		**0.042**
A	104 (20.8)	248 (25.0)	1		1	
B	236 (47.2)	430 (43.3)	1.49 (1.08–2.06)		1.51 (1.09–2.10)	
C	160 (32.0)	314 (31.7)	1.29 (0.93–1.78)		1.21 (0.87–1.70)	
AIDS, *n* (%)				0.378		
*N*	500	992				
Yes	96 (19.2)	174 (17.5)	1.15 (0.85–1.55)			
Current ART, *N* (%)				0.0887		0.1358
TDF/TAF‐based regimen	237 (47.3)	514 (51.8)	1		1	
Other regimen	263 (52.7)	478 (48.2)	1.21 (0.97–1.50)		1.19 (0.95–1.49)	

*Note*: Number of missing values: Weight (*n* = 35), Height (*n* = 115), BMI (*n* = 124), Time since HIV diagnosis (*n* = 10), CD4 nadir (*n* = 52), CD4 count (*n* = 11), CD4% (*n* = 22), CD4/CD8 ratio (*n* = 103), HIV viral load (*n* = 9), CDC disease stage (*n* = 26) and current ART (*n* = 9). Multiple imputation using Chained Equations approach (MICE) were used to fill in missing data. Ten imputations (*M* = 10) were chosen to obtain valid inference and reduce sampling variability resulting from the imputation process. Analyses were run on each of the 10 data sets, and the results were combined with Rubin's rules. Conditional logistic regression model was used to identify factor associated with COVID infection in PLWH. Bold indicates statistically significant values (*p* < 0.05).

Abbreviations: AIDS, acquired immune deficiency syndrome; ART, antiretroviral therapy; BMI, body mass index; CDC, Centers for Disease Control and Prevention; CI, confidence interval; IQR, interquartile range; OR, odds ratio; PLWH, people living with HIV.

## DISCUSSION

Our study revealed that people living with HIV and COVID‐19 have higher rates of several comorbidities, including CKD, as compared to HUC, but a well‐controlled HIV infection was not identified as an independent risk factor for severe COVID‐19 outcomes (critical care admission, palliative care discharge, death); although associations with a longer length of hospital and ICU stay were found. In their 2023 systematic review, Hanson et al. conclude that, according to the literature, morbidity and mortality associated with SARS‐CoV‐2 infection in people living with HIV is complex and that there is no clear consensus thus far [24]. Whilst two systematic reviews and eight individual studies found an increased rate of mortality, hospitalizations and/or severe COVID‐19 outcomes in people living with HIV co‐infected with SARS‐CoV‐2, the other five systematic reviews and six individual studies concluded people living with HIV were not at an increased risk compared to patients without HIV [24]. In a more recent systematic review [22] published in 2024 and evaluating the severity and outcome of COVID‐19 in people living with HIV with COVID‐19 and COVID‐19 controls, the authors concluded that people living with HIV and HIV‐negative individuals showed comparable rates and intensity of COVID‐19. In view of these open questions, our results add important novel evidence based on a solid study design to address this relevant question.

In our study, CKD and multi‐morbidity also stand out as significant risk factors for COVID‐19 in people living with HIV compared to people living with HIV with no COVID‐19. Interestingly, instead of CD4 T cell counts or CD4/CD8 T cell ratio, which are routine immunological surrogate markers for HIV disease stage and immune function, we identified CDC disease stage and detectable HIV‐RNA as risk factors for COVID‐19 infection [25]. The influence of comorbidities on COVID‐19 outcomes has been recognized since the earliest days of the pandemic. Understanding the impact of comorbidity in COVID‐19 facilitates prioritization for interventions (preventive measures, vaccination and early treatment) and it may deepen understanding of the underlying biology of the disease [26]. In the ISARIC4C study [27] more than three‐quarters of hospitalized patients with COVID‐19 had at least one comorbidity, and patients with cardiac disease, pulmonary disease, CKD, obesity, cancer, chronic neurological disorders, dementia and/or liver disease had an increased risk of in‐hospital mortality. Among more than 1700 participants in the UK Biobank with severe SARS‐CoV‐2 infection, about 25% had multi‐morbidity, defined from a list of 12 comorbidities [28]. The combination of stroke and hypertension was most prevalent, and the combination of CKD and diabetes was associated with the highest risk of severe COVID‐19 (OR 4.93; 95% CI, 3.36–7.22). In a UK cohort study [29] using primary‐care data, 15 comorbidity groupings were evaluated together with BMI. The most common comorbidity was hypertension (34.3%), followed by asthma (15.9%) and diabetes (9.9%). However, in age‐ and sex‐adjusted regression models, all comorbidity groups were associated with increased risk of death from COVID‐19; the greatest risk was found in organ‐transplant recipients (HR 6.00; 95% CI, 4.73–7.61) and in those with CKD (HR 3.48 [3.23–3.75]). Interestingly, the magnitude of association was greater in analyses restricted to the earlier pandemic period [26]. We also found some interesting differences in the treatment regimens against COVID‐19 between people living with HIV and HUC since the former received glucocorticoids and anti‐IL 6 inhibitors less often but hydroxychloroquine more often. We can only speculate whether the known HIV status led to more hesitation to treat COVID‐19 in people living with HIV with further immunosuppressive medication.

Depending on the study design and samples size, there are conflicting reports on risk‐factors for COVID‐19 and disease outcomes in people living with HIV. In line with our study, higher rates of comorbidities in people living with HIV and COVID‐19 as compared to matched controls were reported by others [11], and these included COPD, cirrhosis and a history of cancer diagnosis. People living with HIV and uninfected controls had similar COVID‐19 severity on admission as measured by oxygen supplementation requirements but comparable COVID‐19 disease outcomes. A review [23] of 30 studies reported risk factors associated with severity of diseases (e.g., older age, higher BMI, male sex, ethnicity, obesity, smoking, CKD, diabetes, cardiovascular disease, lung cancer, African American, high viral load, low CD4 T cell count, discontinued ART usage and some ART regimens), but very few of these risk factors were confirmed in other studies. Also, 20 studies indicated that clinical presentations among the people living with HIV and COVID‐19 were the same as the general population therefore there was low risk of disease severity [23]. Whilst our study confirms that comorbidities are more prevalent in people living with HIV with COVID‐19 in general (e.g., higher BMI, peripheral vascular disease, dementia, history of pneumonia and liver disease), we provide additional evidence for higher BMI, COPD and HIV disease stage as risk factors for COVID‐19 infection in people living with HIV. In this regard, our study presents for the first time high‐level evidence for CKD being a particular risk factor in people living with HIV, since this condition was significantly more prevalent in people living with HIV with COVID‐19 compared to both control groups. In addition, people living with HIV and COVID‐19 showed a trend to more kidney replacement therapy. Whilst CKD and dialysis are important risk factors for severe COVID‐19 in general [29], especially when combined with other morbidities [29], and COVID‐19 leads to short and long term kidney [30] damage, thus far only a small study indicated chronic kidney dysfunction as potential risk factor in people living with HIV [31]. In contrast, we were surprised to see that the rate of diabetes in people living with HIV and COVID‐19 was significantly lower as compared to COVID‐19 patients without HIV. Whilst an association between diabetes and life‐threatening disease in hospitalized people with COVID‐19 has been described [27] since early in the pandemic, type 2 diabetes often co‐occurs with obesity and cardiovascular disease and is more prevalent in older individuals. Today there seems to be more evidence that type 2 diabetes itself is not causally associated with adverse outcomes in COVID‐19, in contrast to the closely related trait of obesity [32, 33]. Finally, by comparing people living with HIV and COVID‐19 and HUC, we are also evaluating people with and without HIV, independently of COVID‐19 status and this could lead to confounders. Data on the prevalence of conditions such as CKD in cohorts of people living with HIV and seronegative controls matched for age, sex and ethnicity are very limited. The COCOMO Study, for example, provides evidence for a higher prevalence of CKD in people living with HIV, but no increased rates of type 2 diabetes [34]. Therefore, the identification of CKD in our cohort could partially reflect a higher underlying prevalence of CKD in people living with HIV. The same may apply to conditions such as prior pneumonia or liver disease. In contrast, cardiovascular disease (CVD)—which is known to be more prevalent in people living with HIV and a risk factor for severe COVID‐19—did not emerge as a significant risk factor in our analysis. Conversely, type 2 diabetes, another known risk factor for severe COVID‐19, was significantly less common among people living with HIV.

When we started our study, several comorbidities were already described as general risk factors for COVID‐19 and disease courses, which encouraged us to collect many comorbidities for our analysis. The question arises, whether the higher prevalence we found for some conditions in people living with HIV and COVID‐19 is interrelated to the higher pre‐existing comorbidities in people living with HIV in general or associated with COVID‐19 disease? Arguments for the former scenario come from a study analysing the hospital admissions [35]. The authors observed the greatest comorbidity disparities between HIV‐positive and HIV‐negative admissions for mild liver disease (prevalence ratio [PR], 4.9; 95% CI, 4.8–5.1), moderate or severe liver disease (PR, 2.2; 95% CI, 2.0–2.4) and chronic pulmonary disease (PR, 1.8; 95% CI, 1.8–1.8) and these conditions are two among others that we found more often in people living with HIV and COVID‐19. Yet, people living with HIV and COVID‐19 had higher rates of the above‐mentioned comorbidities despite the most likely above‐normal comorbidity rates in the COVID‐19 controls. It is also important to consider that the significant association of a very broad range of comorbidities with hospitalization and death is consistent across not only studies of COVID‐19, but also a range of other respiratory and systemic illnesses. This, together with the strong, consistent signal that multi‐morbidity is the biggest risk factor for severe COVID‐19, suggests that the impact of these comorbidities is not specific to COVID‐19, or even to respiratory disease [26].

We would like to highlight some unique qualities in our study design, which we consider advantageous as compared to previous reports about HIV and COVID‐19. First, the large multi‐centre design (21 sites and five countries) ensured representative data collection of abundant case numbers (people living with HIV and COVID‐19) with matched people living with HIV controls recruited from the same sites and regions. This enabled us to integrate the distinct control group of people living with HIV without COVID‐19. Second, we included important information on HIV disease stage including current CD4 T cell count, HIV‐RNA and antiretroviral therapy. Third, we were able to control for age, sex and ethnicity as known risk factors for the COVID‐19 disease course. We also matched for the time of COVID‐19 diagnosis to contain variability due to, for example, constraints in health care services during the early phase of the pandemic and matched for place of COVID‐19 diagnosis (inpatient/ambulatory), which otherwise could have had an important influence on the primary and secondary outcome measures. Fourth, we measured 17 different comorbidities, most of which have known impact on either SARS‐CoV‐2 infection and/or COVID‐19 disease course and included the CCI as an established score to estimate mortality risk in people with multi‐morbidity. Fifth, when defining inclusion criteria and our composite primary endpoint, we deliberately avoided hospitalization as a component. Associations reported between comorbidities and outcomes in studies of populations of hospitalized patients with COVID‐19 could be biased owing to the criteria for entry into the study (that is, requirement for hospitalization) being causally associated with both the comorbidity [36] and the outcome. Studies undertaken in populations that are not restricted to hospitalized individuals are less likely to report associations affected by this bias [26]. Caution is also needed when severity is defined by health‐service use or intervention (that is, hospitalization [37], critical care admission or respiratory support), as the presence of a comorbidity (e.g., HIV infection) might influence clinical decision making, either lowering or increasing the threshold for hospitalization or provision of organ support. It is possible that the extreme capacity strain in many areas during the early peak of the COVID‐19 pandemic may have augmented this effect with relevance to people with known HIV status [26]. To strengthen in‐hospital related severe COVID‐19 disease outcomes, we captured important secondary outcomes including number of ventilator‐free days, length of extracorporeal membrane oxygenation (ECMO) and need for kidney replacement therapy.

Our study also has some limitations. We did not assess COVID‐19 related parameters like baseline SARS‐CoV‐2 viral load, which can have an impact on the COVID‐19 disease course and most people living with HIV had good immune function with median CD4 T cell counts above 600 cells per mL [38]. We do not have complete biomedical data for all individuals, and we were unable to assess, for example, socioeconomic factors, which together with ethnicity and environment influence the likelihood of infection and subsequent outcomes [26]. Whilst several sites from European countries participated in this study, we are not fully representative of the entire European region. One could consider the fact that our study period is restricted to the time before the start of widespread COVID‐19 vaccination as a limitation. However, since vaccinations and hybrid immunity significantly influenced the COVID‐19 disease course, we expected that the impact of, for example, number of COVID‐19 vaccinations, various vaccine schedules and infections with different SARS‐CoV‐2 variants would have been important confounders and difficult to control for [39]. Thus, we can only make conclusions about the initial phase of the corona pandemic when SARS‐CoV‐2 hit people living with HIV with a naïve immune system for the infection. When comparing people living with HIV who have and have not had COVID‐19, it is important to consider that those who seek testing and report infection may differ in baseline characteristics from those who do not, potentially introducing bias. Also, self‐reporting is unreliable and might influence the results. We believe that recruiting people living with HIV as controls from the same sites or outpatient clinics where the people living with HIV and COVID‐19 cases were diagnosed and treated was the most practical way to reduce confounding effects related to variations in pandemic waves, healthcare systems, testing strategies or sociodemographic factors.

In summary, based on improved evidence, our study did not identify a well‐controlled HIV infection as a significant risk factor for severe COVID‐19 overall, but we found associations with a longer length of hospital and ICU stay. We confirmed several risk factors for COVID‐19 in people living with HIV, among which our study identified CKD and multi‐morbidity of particular relevance.

## AUTHOR CONTRIBUTIONS

*Study design*: Georg M. N. Behrens, Anton Pozniak and Lambert Assoumou. *Data collection and curation*: Stephane De Wit, Rona MacDonald, Nathalie de Castro, Casper Rokx, Holly Middleditch, Margaret Johnson, Jose Luis Casado, Jose Ramon Arribas, Jose‐Ramon Blanco, Caroline Eteve‐Pitsaer, Carl Fletcher. *Data analysis*: Aliou Baldé and Lambert Assoumou. *Data interpretation*: Lambert Assoumou, Georg M. N. Behrens, Anton Pozniak and Esteban Martinez. *Writing*: Georg M. N. Behrens, Anton Pozniak and Esteban Martinez with comments from all authors. Georg M.N. Behrens and Lambert Assoumou have directly accessed and verified the underlying data reported in the manuscript.

## FUNDING INFORMATION

This work was supported by an investigator‐sponsored research grant (ISR) from Gilead (IN‐DE‐983‐6076) and the NEAT ID Foundation.

## CONFLICT OF INTEREST STATEMENT

Georg M. N. Behrens has received honoraria for lectures and advisory boards from Gilead, ViiV Healthcare, MSD, Janssen, Novartis, EUROIMMUN, Moderna and Pfizer. He received funding for research from Novartis to his institution unrelated to this work. Esteban Martinez has received honoraria for lectures or advisory boards from Gilead, Janssen, MSD and ViiV, and his institution has received research grants from MSD and ViiV. Anton Pozniak has received honoraria for lectures and advisory boards from Gilead, ViiV Healthcare and MSD. Casper Rokx declares honoraria for advisory boards and a research grant for investigator‐initiated studies from Gilead and ViiV Healthcare paid to his institution. All other authors declare no conflicts of interest.

## ETHICS STATEMENT

The NAET ID HIV CoCo Study (NCT05481216) and the analysis conducted for this article were approved by the relevant ethics committees in the United Kingdom, Spain, Belgium and the Netherlands.

## Supporting information


**Data S1.** Supporting information.

## Data Availability

All requests for raw and analysed data that underly the results reported in this article should be sent to the corresponding author and will be reviewed within 4 weeks by the NEAT ID HIV CoCo Study Team to determine whether the request is subject to confidentiality and data protection obligations. Data that can be shared will be released via a material transfer agreement.
