# Supplementary material for: Morbidity and risk factors of COVID‐19 in people with HIV‐1 in Europe: A combined observational cohort and nested case–control study
Source: HIV Med. 2025 Aug 7;26(11):1657–72. doi: 10.1111/hiv.70094 (PMC12579890; doi:10.1111/hiv.70094)
Supplement: Supplementary file 1 — Data S1. Supporting information. [file HIV-26-1657-s001.docx]

**Supplementary Material**

**Morbidity and Risk Factors of COVID-19 in People with HIV-1 in Europe: A combined observational cohort and nested case-control study**

Georg M.N. Behrens, Lambert Assoumou, Stephane De Wit, Rona MacDonald, Nathalie de Castro, Casper Rokx, Holly Middleditch, Margaret Johnson, Jose Luis Casado, Jose Ramon Arribas, Jose-Ramon Blanco, Carl Fletcher, Annie Duffy, Caroline Eteve-Pitsaer, Aliou Baldé, Anton Pozniak, Esteban Martinez, on behalf of the HIV CoCo Study Group

**Methods**

*Statistical Analysis*

For an 80% power, a 5% type I error and an expected severity of COVID-19 infection of 15% HUC individuals, 500 PLWH and COVID-19 infection (exposed to HIV) and 500-1500 HUC and COVID-19 infection (unexposed to HIV) were needed to detect a 1.436 to 1.520-fold increase in hazard rate in PLWH and COVID-19 infection relative to HUC and COVID-19 infection. For the search for factors associated with SARS-CoV-2 infection in PLWH, the recommended sample size estimation for multivariable logistic regression models was n = 100 + 50i where i refers to number of independent variables in the final model[24]. With 1500 PLWH (500 with COVID-19 and 1000 controls without COVID-19), we would be able to study 28 parameters with sufficient power to produce statistics that are nearly representative of the true values in the targeted population. The power calculation was made using the statistical software package nQuery Advanced, with the two samples Log-Rank test module (Version 8.6.1.0).

The population analyzed for COVID-19 severity consisted of all HIV-exposed individuals (PLWH and COVID-19) and a matched unexposed HUC group with COVID-19. All exposed individuals without matched unexposed controls were excluded from the analysis. The characteristics of exposed and unexposed individuals to HIV were compared with a McNemar test for categorical variables and Wilcoxon paired test for continuous variables.

Time-to-event methods, including Kaplan–Meier estimates and Cox proportional-hazards models, were used to analyze the occurrence of a severe form of COVID-19 between PLWH and COVID-19 and HUC and COVID-19. Unadjusted and adjusted hazard ratios (HR) were calculated using Cox proportional hazard model stratified on each pair to assess whether HIV-infection was associated with the severity of COVID-19. The following factors associated with COVID-19 severity were accounted for in multivariable models: BMI, dementia, peripheral vascular disease, history of pneumonia, connective tissue disease, liver disease, diabetes, chronic kidney disease, glucocorticoids, antibiotics, hydroxychloroquine, tocilizumab, and anti-IL6 inhibitors. Similar analyses were performed for each component of the composite primary endpoint.

The proportion of hospitalized participants, discharged from hospital and duration of hospitalization, discharged from ICU and length stay in ICU and the number of ventilator-free days between PLWH and COVID-19 and HUC and COVID-19 was estimated using competing risk regression with death as a competing event. Unadjusted and adjusted subdistribution hazard ratio (sHR) were calculated using the Fine-Gray proportional hazards model for subdistribution stratified on each matched pair.

The proportion of participants requiring kidney replacement therapy was estimated by dividing the number of participants requiring kidney replacement by the total number of participants per group and was compared between the two groups using stratified logistic regression analysis on each matched pair.

Generalized estimating equations (GEE) with a negative binomial distribution, log link functions and independent covariance structure were used to compare the mean difference between PLWH and COVID-19 and HUC and COVID-19 in CCI, ACCI, estimated risk of 42-day mortality after COVID-19, inflammatory markers, blood cells count and all other biological parameters at COVID-19 diagnosis. The 95% confidence intervals of the mean difference between the two groups were obtained by bootstrap.

To identify risk factors (e.g. CD4 T cell nadir, current CD4 T cell count, co-morbidities) for COVID-19 within the group of PLWH, conditional logistic regression models accounting for case-control matching were used. The analysis population consisted of PLWH with COVID-19 and matched PLWH without COVID-19. The following variables were tested : Obesity (BMI>30 kg/m^2^); Comorbidities [Myocardial Infarction (Heart Attack); Congestive Heart Failure; Peripheral Vascular Disease; Cerebrovascular Accident (Stroke) or Transient Ischemic Attack; Dementia; Chronic Obstructive Pulmonary Disease; History of Pneumonia; Connective Tissue Disease; Peptic Ulcer Disease; Liver Disease; Diabetes; Hemiplegia; Paralysis of Arm(s) or Leg(s); Chronic Kidney Disease; Current or History of Cancer; Leukemia; Lymphoma; AIDS]; HIV information [time since HIV diagnosis (years); current ART; CDC disease stage; prior AIDS event; CD4 cell nadir; last CD4 cell count/HIV-RNA before COVID-19 diagnosis (including CD4 T cell percentage and CD4/CD8 ratio)]. For continuous variables, the decision to treat the variable as a continuous or categorical variable (as tertile) were based on the lowest Akaike information criterion value for the corresponding univariable conditional logistic regression analysis. Variables with univariable p-value <0.20 were retained for the multivariable analysis. Because some of the variables for the multivariable analysis had missing values, multiple imputations using Chained Equations approach (MICE) were used to fill in missing data. Ten imputations (M=10) were chosen to obtain valid inference and reduce sampling variability resulting from the imputation process. Analyses were run on each of the 10 data sets, and the results were combined with Rubin’s rules.

Analyses were conducted with SAS software version 9.4 (SAS Statistical Institute, Cary, North Carolina). All statistical tests were 2-tailed, with α = 0.05.

**Suppl. Table S1. Laboratory parameter**

|  |  | **Participants** | |  |
| --- | --- | --- | --- | --- |
| **Biological parameters** |  | **PLWH and COVID-19** | **HUC and COVID-19,** | **P-value**^1^ |
|  |  | **N=486** | **N=1106** |  |
| **Platelet count (10^9^/L)** |  |  |  | **0.023** |
|  | N | 180 | 185 |  |
|  | Median (IQR) | 202 (159-263) | 217 (165-283) |  |
|  | [Range] | [34-522] | [12-709] |  |
|  | Mean (Std) | 212 (87.9) | 237 (105.5) |  |
| **RBC Count (10^12^/L)** |  |  |  |  |
|  | N | 172 | 177 | **0.002** |
|  | Median (IQR) | 4.5 (3.9-4.9) | 4.7 (4.3-5.0) |  |
|  | [Range] | [2.0-6.6] | [1.6-6.4] |  |
|  | Mean (Std) | 4.4 (0.8) | 4.6 (0.7) |  |
| **WBC count (10^9^/L)** |  |  |  | 0.767 |
|  | N | 178 | 183 |  |
|  | Median (IQR) | 6.2 (5.0-8.7) | 6.4 (4.8-8.8) |  |
|  | [Range] | [1.7-22.2] | [0.03-29.2] |  |
|  | Mean (Std) | 7.2 (3.5) | 7.4 (4.1) |  |
| **Haemoglobin (g/dL)** |  |  |  | 0.274 |
|  | N | 178 | 183 |  |
|  | Median (IQR) | 13.3 (11.6-14.5) | 13.5 (12.2-14.4) |  |
|  | [Range] | [5.0-17.4] | [4.3-17.9] |  |
|  | Mean (Std) | 13.0 (2.3) | 13.1 (2.2) |  |
| **Haematocrit (L/L)** |  |  |  | 0.772 |
|  | N | 173 | 178 |  |
|  | Median (IQR) | 3.6 (0.4-4.3) | 3.6 (0.4-4.2) |  |
|  | [Range] | [0.2-34.0] | [0.2-39.0] |  |
|  | Mean (Std) | 2.8 (3.0) | 2.8 (3.3) |  |
| **MCV (fL)** |  |  |  | **<0.001** |
|  | N | 176 | 181 |  |
|  | Median (IQR) | 92.0 (87.1-96.0) | 88.1 (84.9-90.9) |  |
|  | [Range] | [68.8-109.0] | [11.7-118.0] |  |
|  | Mean (Std) | 91.4 (7.3) | 87.3 (8.6) |  |
| **MCH (pg)** |  |  |  | **<0.001** |
|  | N | 171 | 175 |  |
|  | Median (IQR) | 30.6 (28.5-32.0) | 29.2 (27.9-30.7) |  |
|  | [Range] | [19.1-37.1] | [20.7-37.4] |  |
|  | Mean (Std) | 30.2 (2.8) | 29.1 (2.4) |  |
| **Neutrophils (10^9^/L)** |  |  |  | 0.214 |
|  | N | 165 | 169 |  |
|  | Median (IQR) | 4.5 (3.1-6.6) | 4.9 (3.2-7.0) |  |
|  | [Range] | [0.8-102] | [0.01-85.3] |  |
|  | Mean (Std) | 7.0 (11.9) | 8.3 (14.3) |  |
| **Lymphocytes (10^9^/L)** |  |  |  | **<0.001** |
|  | N | 168 | 171 |  |
|  | Median (IQR) | 1.3 (0.9-1.9) | 1.1 (0.7-1.5) |  |
|  | [Range] | [0.0-36.1] | [0.01-29.0] |  |
|  | Mean (Std) | 2.0 (3.9) | 1.8 (3.2) |  |
| **Monocytes (10^9^/L)** |  |  |  | 0.079 |
|  | N | 167 | 170 |  |
|  | Median (IQR) | 0.5 (0.3-0.7) | 0.4 (0.3-0.6) |  |
|  | [Range] | [0.0-38.0] | [0.01-10.0] |  |
|  | Mean (Std) | 1.0 (3.3) | 0.7 (1.1) |  |
| **Eosinophils (10^9^/L)** |  |  |  | 0.310 |
|  | N | 162 | 166 |  |
|  | Median (IQR) | 0.01 (0.0-0.1) | 0.01 (0.0-0.1) |  |
|  | [Range] | [0.0-0.7] | [0.0-6.0] |  |
|  | Mean (Std) | 0.07 (0.12) | 0.13 (0.59) |  |
| **Basophils (10^9^/L)** |  |  |  | 0.162 |
|  | N | 160 | 163 |  |
|  | Median (IQR) | 0.01 (0.0-0.03) | 0.01 (0.0-0.03) |  |
|  | [Range] | [0.0-1.7] | [0.0-2.0] |  |
|  | Mean (Std) | 0.05 (0.17) | 0.04 (0.16) |  |
| **ALT (U/L)** |  |  |  | **0.009** |
|  | N | 156 | 158 |  |
|  | Median (IQR) | 27.0 (18.0-45.0) | 30.5 (21.0-57.0) |  |
|  | [Range] | [5.1-963.0] | [9.0-309.0)] |  |
|  | Mean (Std) | 42.0 (78.9) | 47.4 (44.7) |  |
| **AST (U/L)** |  |  |  | 0.258 |
|  | N | 122 | 124 |  |
|  | Median (IQR) | 37.0 (24.0-50.5) | 40.0 (26.0-60.0) |  |
|  | [Range] | [5.8-1284.0] | [11.0-350.0] |  |
|  | Mean (Std) | 55.2 (118.4) | 54.0 (50.5) |  |
| **Total bilirubin (mg/dL)** |  |  |  | 0.613 |
|  | N | 151 | 152 |  |
|  | Median (IQR) | 0.5 (0.3-0.7) | 0.5 (0.4-0.7) |  |
|  | [Range] | [0.2-7.8] | [0.1-9.6] |  |
|  | Mean (Std) | 0.7 (0.9) | 0.7 (0.9) |  |
| **Urea (mg/dL)** |  |  |  | 0.092 |
|  | N | 144 | 145 |  |
|  | Median (IQR) | 31.8 (23.0-46.2) | 31.2 (23.0-46.0) |  |
|  | [Range] | [4.0-508.7] | [1.8-213.8] |  |
|  | Mean (Std) | 52.6 (64.0) | 39.7 (30.2) |  |
| **Serum creatinine (mg/dL)** |  |  |  | **0.020** |
|  | N | 173 | 176 |  |
|  | Median (IQR) | 1.0 (0.8-1.2) | 0.9 (0.8-1.1) |  |
|  | [Range] | [0.3-25.8] | [0.3-10.7] |  |
|  | Mean (Std) | 1.8 (3.3) | 1.1 (1.1) |  |
| **Calcium (mg/dL)** |  |  |  | 0.727 |
|  | N | 96 | 96 |  |
|  | Median (IQR) | 2.3 (2.1-8.6) | 2.3 (2.2-8.2) |  |
|  | [Range] | [1.8-9.9] | [1.8-88.0] |  |
|  | Mean (Std) | 4.9 (3.2) | 5.7 (9.0) |  |
| **glucose (mmol/L)** |  |  |  | 0.264 |
|  | N | 94 | 98 |  |
|  | Median (IQR) | 5.7 (5.1-7.4) | 5.8 (5.1-7.6) |  |
|  | [Range] | [3.9-25.1] | [4.0-44.5] |  |
|  | Mean (Std) | 7.0 (3.6) | 7.4 (5.1) |  |
| **HbA1C levels (mmol/mol)** |  |  |  | 0.461 |
|  | N | 4 | 4 |  |
|  | Median (IQR) | 45.5 (39.0-82.0) | 80.5 (43.0-115.3) |  |
|  | [Range] | [36.0-115.0] | [42.0-193.6] |  |
|  | Mean (Std) | 60.5 (36.7) | 99.2 (72.0) |  |
| **total cholesterol (mmol/L)** |  |  |  | **0.041** |
|  | N | 31 | 31 |  |
|  | Median (IQR) | 4.5 (3.7-5.0) | 3.6 (3.0-4.9) |  |
|  | [Range] | [2.4-6.0] | [1.5-6.7] |  |
|  | Mean (Std) | 4.4 (0.9) | 3.8 (1.3) |  |
| **Triglycerides (mmol/L)** |  |  |  | 0.669 |
|  | N | 34 | 34 |  |
|  | Median (IQR) | 1.3 (1.0-1.7) | 1.5 (0.9-2.0) |  |
|  | [Range] | [0.5-4.3] | [0.6-5.5] |  |
|  | Mean (Std) | 1.6 (0.9) | 1.9 (1.3) |  |
| **CRP (mg/L)** |  |  |  | 0.898 |
|  | N | 129 | 131 |  |
|  | Median (IQR) | 7.2 (2.9-15.6) | 9.9 (3.2-15.5) |  |
|  | [Range] | [0.09-164.8] | [0.0-221.0] |  |
|  | Mean (Std) | 12.6 (18.7) | 13.6 (21.8) |  |
| **D-dimer (ng/mL)** |  |  |  | 0.411 |
|  | N | 87 | 87 |  |
|  | Median (IQR) | 843 (440-1780) | 700 (340-1596) |  |
|  | [Range] | [143-18025] | [149-11102] |  |
|  | Mean (Std) | 1653.8 (2452.7) | 1561.9 (2299.8) |  |
| **Ferritin (ng/mL)** |  |  |  | 0.375 |
|  | N | 58 | 59 |  |
|  | Median (IQR) | 455 (149-1376) | 845 (434-1381) |  |
|  | [Range] | [17-7824] | [35-7185] |  |
|  | Mean (Std) | 1039.5 (1398.8) | 1113.0 (1143.6) |  |
| **Lactate dehydrogenase, LDH (U/L)** |  |  |  | **0.038** |
|  | N | 86 | 86 |  |
|  | Median (IQR) | 308 (227-423) | 358 (268-441) |  |
|  | [Range] | [129-1214] | [164-1900] |  |
|  | Mean (Std) | 357.5 (205.3) | 412.2 (237.1) |  |
|  | | | | |

^1^ P-value were calculated using McNemar test for categorical variable and Wilcoxon paired test for continuous variables HUC, HIV-uninfected controls

**Suppl. Table S2. COVID-19 treatment**

|  | **Participant** | |  |
| --- | --- | --- | --- |
| **Drug treatment for COVID-19, N= (%)** | **PLWH and COVID-19** | **HUC and COVID-19** | **P value** |
| Glucocorticoids | 32/109 (29.4) | 53/109 (48.6) | **0.002** |
| Azithromycin | 39/109 (35.6) | 46/109 (42.2) | 0.151 |
| Other antibiotics | 39/109 (35.8) | 44/109 (40.4) | 0.338 |
| Hydroxychloroquine | 62/126 (49.2) | 49/129 (38.0) | **0.007** |
| Remdesivir | 9/126 (7.1) | 14/129 (10.9) | 0.177 |
| Tocilizumab | 5/126 (4.0) | 3/129 (2.3) | 0.424 |
| monoclonal antibodies against SARS-CoV2 | 1/126 (0.8) | 7/129 (5.4) | 0.069 |
| Reconvalescent plasma | 3/126 (2.4) | 1/129 (0.8) | 0.341 |
| Anti-IL1 inhibitors | 2/126 (1.6) | 1/129 (0.8) | 0.571 |
| Anti-IL6 inhibitors | 8/126 (6.4) | 20/129 (15.5) | **0.010** |
| Lopinavir/ritonavir | 25/126 (19.8) | 36/129 (27.9) | 0.054 |
| Anticoagulants | 13/109 (11.9) | 18/109 (16.5) | 0.177 |
| Others | 18/109 (16.5) | 33/109 (30.3) | **0.008** |

HUC, HIV-uninfected controls

**Suppl. Table S3. Secondary outcomes of PLWH and COVID-19 versus COVID-19**

|  | **PLWH and COVID-19** | **HUC and COVID-19** | **Crude measure of association (95% CI)** | **Adjusted measure of association (95% CI)*** |
| --- | --- | --- | --- | --- |
|  | **N=486** | **N=1106** |  |  |
| **Hospitalisation** |  |  |  |  |
| Number of hospitalized patients | 200 | 208 |  |  |
| Number of deaths before hospitalisation (competing risk) | 1 | 1 |  |  |
| Cumulative incidence of inpatients – % (95% CI) | 23.2  (5.4-99.4) | 21.7  (5.3-88.5) | sHR (95% CI)  1.08 (1.01-1.15) | sHR (95% CI)  0.98 (0.89-1.07) |
| Number of discharge | 155 | 168 |  |  |
| Number of deaths in hospital (competing risk) | 23 | 22 |  |  |
| Cumulative incidence of discharge from hospital – % (95% CI) | 60.3  (60.3-60.4) | 63.2  (63.2-63.3) | sHR (95% CI)  0.93 (0.76-1.13) | sHR (95% CI)  0.63 (0.48-0.84) |
| Median length of stay in hospital (95% CI) - days | 11  (9-12) | 9  (8-11) |  |  |
| **ICU discharge** |  |  |  |  |
| Number of participants | 19 | 19 |  |  |
| Number of discharge from ICU | 14 | 15 |  |  |
| Number of deaths in ICU (competing risk) | 2 | 4 |  |  |
| Cumulative incidence of discharge from ICU – % (95% CI) | 72.6  (65.8-80.1) | 95.5  (92.9-98.3 | sHR (95% CI)  0.42 (0.19-0.93) | sHR (95% CI)  0.06 (0.01-0.34) |
| Median length of stay on ICU (95% CI) - days | 18  (11-33) | 7  (4-21) |  |  |
| **Number of ventilator-free days (VFDs)** |  |  |  |  |
| Number of participants | 7 | 7 |  |  |
| Number of extubation | 4 | 4 |  |  |
| Number of deaths during invasive ventilation (competing risk) | 2 | 3 |  |  |
| Cumulative incidence of extubation – % (95% CI) | 75.3  (64.5-88.0) | 87.8  (77.6-99.2) | **s**HR (95% CI)  0.67 (0.18-2.42) | **s**HR (95% CI)  0.60 (0.15-2.42) |
| Median duration on Mechanical ventilation (95% CI) - days | 18  (8-42) | 19  (3-34) |  |  |
| **Length of extracorporeal membrane oxygenation (ECMO)** |  |  |  |  |
| Number of participants | 0 | 0 |  |  |
| Number of pts no longer receive ECMO | 0 | 0 |  |  |
| Number of deaths during ECMO | 0 | 0 |  |  |
| Cumulative incidence of end of ECMO – % (95% CI) | 0 | 0 | na | na |
| Median length of ECMO (95% CI) - days | 0 | 0 | na | na |
| **Need for kidney replacement therapy** |  |  |  |  |
| Number of participants | 129 | 129 |  |  |
| Number of pts requiring renal replacement therapy | 13 | 6 |  |  |
| Proportion of participants requiring renal replacement therapy – % (95% CI) | 10.1  (5.5-16.6) | 4.6  (1.7-9.9) | OR (95% CI)  2.17 (0.82-5.70) | OR (95% CI)  1.81 (0.54-6.02) |
| **Charlson Comorbidity Index (CCI)** |  |  |  |  |
| Number of participants | 170 | 170 |  |  |
| Mean (95% CI) | 1.61  (1.17-2.04) | 0.25  (0.15-0.35) | Mean difference (95% CI)  1.36 (1.09 – 1.73) | Mean difference (95% CI)  1.38 (0.73 – 2.18) |
| **Age-adjusted CCI (ACCI)** |  |  |  |  |
| Number of participants | 170 | 170 |  |  |
| Mean (95% CI) | 2.35  (1.87-2.83) | 0.99  (0.82-1.16) | Mean difference (95% CI)  1.35 (1.06 – 1.65) | Mean difference (95% CI)  1.62 (1.14 – 2.22) |
| **Estimate risk of 30-day mortality after COVID-19 infection** |  |  |  |  |
| Number of participants | 172 | 172 |  |  |
| Mean (95% CI) | 4.71  (3.76-5.66) | 3.48  (2.85-4.11) | Mean difference (95% CI)  1.22 (0.77 – 1.64) | Mean difference (95% CI)  1.61 (0.40 – 3.05) |

HUC, HIV-uninfected controls
